# Hybrid Minimally Invasive Esophagectomy vs. Open Esophagectomy: A Retrospective Propensity Score Matched Comparison

**DOI:** 10.3390/medicina59030434

**Published:** 2023-02-22

**Authors:** Anna Vincke, Sorin Miftode, Fadl Alfarawan, Maximilian Bockhorn, Nader El-Sourani

**Affiliations:** Department for General and Visceral Surgery, University Hospital Oldenburg, Klinikum Oldenburg AöR, Rahel-Straus-Straße 10, 26133 Oldenburg, Germany

**Keywords:** esophageal cancer, esophagectomy, minimally invasive techniques, morbidity, risk factors, surgical outcome

## Abstract

*Background and Objectives*: Though widely used, only limited data is available that shows the superiority of hybrid minimally-invasive esophagectomy (HMIE) compared to open esophagectomy (OE). The present study aimed to analyze postoperative morbidity, mortality, and compare lengths of hospital stay. *Materials and Methods*: A total of 174 patients underwent Ivor Lewis esophagectomy in our surgical department, of which we retrospectively created a matched population of one hundred (HMIE *n* = 50, OE *n* = 50). Morbidity and mortality data was categorized, analyzed, and risk factor analyzed for complications. *Results*: The oncological results were found to be comparable in both groups. A median of 23.5 lymphnodes were harvested during OE, and 21.0 during HMIE. Negative tumor margins were achieved in 98% of OE and 100% of HMIE. In-hospital mortality rate showed no significant difference between techniques (OE 14.0%, HMIE 4.0%, *p* = 0.160). Hospital (OE Median 23.00 days, HMIE 16.50 days, *p* = 0.004) and ICU stay (OE 5.50 days, HMIE 3.00 days, *p* = 0.003) was significantly shorter after HMIE. The overall complication rate was 50%, but complications in general (OE 70.00%, HMIE 30%, *p* < 0.001) as well as severe complications (Clavien Dindo ≥ III: HMIE 16.0%, OE 48.0%, *p* < 0.001) were significantly more common after OE. In multivariate stepwise regressions the influence of OE proved to be independent for said outcomes. We observed more pulmonary complications in the OE group (46%) compared to HMIE patients (26%). This difference was statistically significant after adjustment for sex, age, BMI, ASA classification, histology, neoadjuvant treatment or not, smoking status, cardiac comorbidities, diabetes mellitus, and alcohol abuse (*p* = 0.019). *Conclusions*: HMIE is a feasible technique that significantly decreases morbidity, while ensuring equivalently good oncological resection compared to OE. HMIE should be performed whenever applicable for patients and surgeons.

## 1. Introduction

The German S3-Guideline for diagnosis and treatment of squamous cell carcinoma and adenocarcinoma of the esophagus recommends any kind of minimally-invasive esophagectomy, including the laparoscopic-thoracotomic hybrid approach (HMIE) as the surgical procedure of choice for surgical treatment of esophageal cancer instead of open esophagectomy (OE) [1]. A few studies showed less postoperative morbidity as well as shorter hospital and intensive care unit (ICU) stay for the total minimally invasive approach (MIC) compared to OE [2,3]. Nevertheless, for some patients or surgical centers MIC cannot be the method of choice. Instead, a 2020 meta-analysis with a total of 3732 patients showed some disadvantages of MIC compared to Ivor Lewis HMIE [4]. Not only was the operating time of MIC significantly higher, but also significantly more anastomotic leakages occurred. Another meta-analysis by Bras et al. showed a similar overall morbidity rate for HMIE and MIC (HMIE 40%, 95% CI: 25–59%; MIC 37%, 95% CI: 32–43%; OR 1.13) [5]. For the alternative HMIE approach, only a few current studies showed superiority regarding morbidity and non-inferiority regarding survival in comparison to the OE technique [6,7]. For example, the MIRO trial by Mariette et al. demonstrated a significantly lower major intraoperative and postoperative morbidity in the first 30 postoperative days after HMIE compared to OE [6]. The length of hospital stay was equal in both groups, and variables like postoperative anemia or need of red blood cells (RBCs) were not reported.

The objective of this retrospective study was to compare the HMIE and OE approaches regarding the aforementioned perioperative and oncological results in the setting of a single high-volume center with a large matched study population. An additional objective was to compare HMIE and OE regarding factors like hospital stay, as this kind of advantage has been shown for the equivalently recommended MIC technique [8].

## 2. Materials and Methods

### 2.1. Study Design and Patients

**Figure 1:**
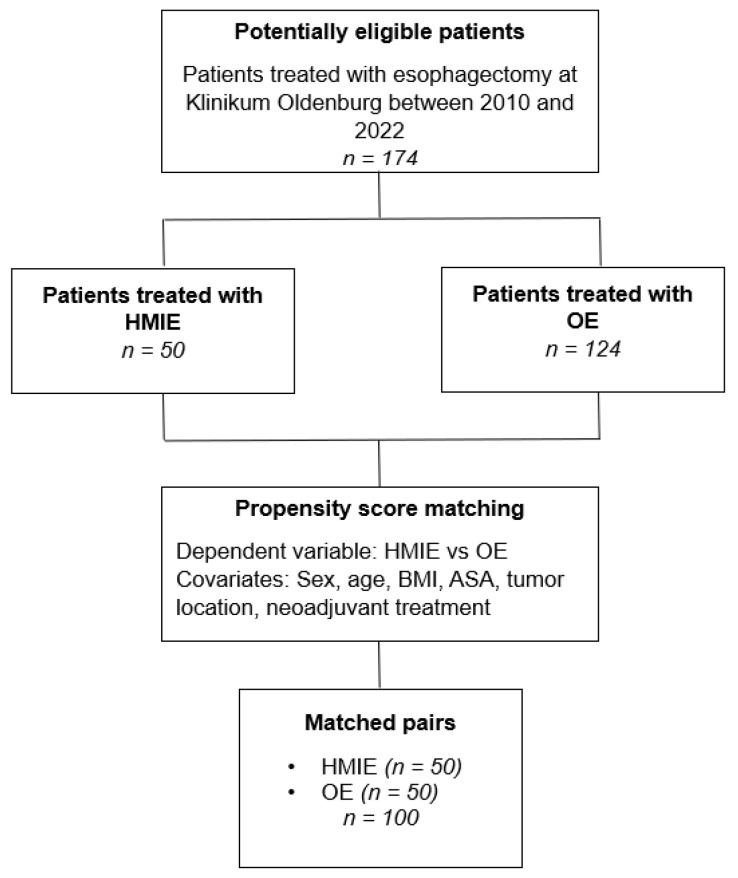
Study design.

In our surgical department 174 patients received an Ivor Lewis esophagectomy for treatment of esophageal cancer from January 2010 until August 2022. HMIE was introduced as a new operating technique for underlying malignancy of the esophagus in 2019. Due to the new implementation, a hybrid approach was favored over MIC. In addition, HMIE is preferred over MIC at our institution, as it seems to be associated with a lower rate of anastomotic leakage [4,9]. This study retrospectively analyzed perioperative morbidity and mortality and the differences between two surgical techniques. HMIE was performed on 50 patients. We paired these with 50 patients treated with OE. All patients with an esophagectomy and reconstruction with gastric pull-up operated on in our surgical department in the aforementioned time span were considered for matching. This study was conducted in accordance with the ethics committee of the University of Oldenburg (AZ 2022-116). A flow chart of the study design is shown in Figure 1.

### 2.2. Preoperative Process

Patients were either referred by outpatient physicians or by the gastroenterology department of our hospital. Before receiving any treatment, patients were evaluated for their suitability for surgery by computed tomography (CT) of the neck, thorax, and abdomen and endoscopic ultrasound (EUS). Furthermore, all patients were discussed in a multidisciplinary tumor board to ensure a stage-related therapy. According to the S3-guideline for diagnosis and treatment of squamous cell carcinoma and adenocarcinoma of the esophagus, patients either received neoadjuvant treatment in the form of radiotherapy, chemotherapy, or radiochemotherapy [1,10,11]. Some patients received no neoadjuvant treatment and others were treated with endoscopic submucosal dissection prior to surgery. After completion of neoadjuvant therapy, restaging including CT and EUS was conducted.

### 2.3. Surgical Techniques

Directly before surgery an epidural catheter was placed in all patients to ensure adequate postoperative analgesia.

**Figure 2:**
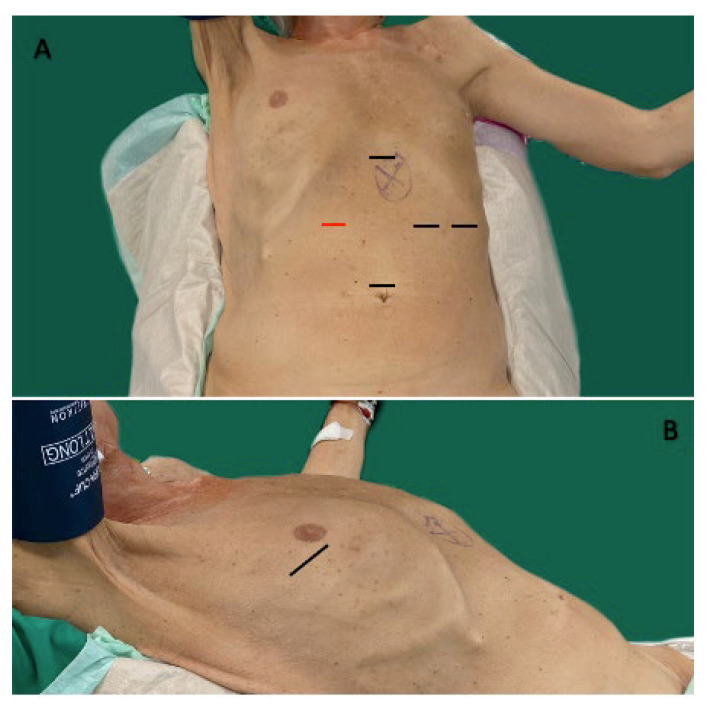
Positioning of the patient and placement of trocars. (**A**): Showing the placement of all five 5 mm–12 mm trocars. Red indicates placement of a 12 mm trocar. (**B**): Showing the positioning of the patient from the right side with the mark indicating the incision for open thoracotomy.

All operations were performed by two surgeons at all times. HMIE was done through a 2-stage approach consisting of a laparoscopic abdominal and a muscle-preserving open right thoracotomy phase. The patient was brought in a supine “left lateral” position in combination with a “beach-chair” position (Figure 2). This way, the 2-stage approach could be performed in one setting without the need for re-positioning.

During the abdominal phase, the first trocar was usually placed supra-umbilically. After induction of a pneumoperitoneum, four additional trocars were placed in a diamond-fashion where the one in the right upper quadrant must be at least a 12 mm port, which is needed for fashioning the gastric conduit (Figure 2). At first, the stomach was mobilized while preserving the gastric arcade and the right gastroepiploic artery and vein. A two-field lymphadenectomy was performed in concordance with the national treating guidelines for esophageal cancer [1]. Here, the left gastric artery and vein were identified, mobilized, and divided between two clips. Following complete mobilisation of the stomach, a 4–6 cm wide gastric conduit was fashioned by sequential firings of 45 mm Endo-GIA™ (Medtronic) cartridges parallel to the greater curvature, whereas the first firing was applied across the lesser curvature just above the pylorus and nearly perpendicular to the greater curvature. Following the complete mobilisation of the stomach and construction of the gastric conduit, an abdominal drain was inserted through the leftmost trocar and placed under the gastric conduit. This marked the end of the abdominal phase.

Following, a muscle-preserving right anterior thoracotomy was performed. The right lung was excluded using a double-lumen tube. The arch of the azygous vein was mobilized and divided using an Endo-GIA™ Curved Tip (Medtronic, Meerbusch, Germany). A routine infracarinal lymphadenectomy was performed. Before fashioning the esophagogastric anastomosis using a 25 mm EEA™ Auto Suture Circular Stapler (Medtronic, Meerbusch, Germany), an intraoperative pathology consultation took place to confirm free resection margins. Finally, two chest drains were placed before closing the thoracotomy—one perianastomotically and one at the base of the lung. All patients were extubated in the OR and transported to the ICU.

### 2.4. Postoperative Management

After surgery, all patients were primarily treated at the ICU and when stable enough, transferred to the intermediate care unit (IMC). Later, they were treated at the surgical ward. Enteral nutrition was standardized in all patients and started within 24 h postoperatively. For the first 4 postoperative days patients received nutrition via an intraoperatively inserted nasoduodenal feeding tube. From the fifth postoperative day, oral feeding was started with fluids and increased depending on clinical condition. Routine examination of the anastomosis was not performed. A combination of CT and upper endoscopy (UE) was only performed if an anastomotic leakage was suspected. In patients with severe complications (i.e., anastomotic leakage) a triple nasojejunal feeding tube was inserted after placing the endoluminal vacuum therapy.

### 2.5. Statistical Analysis

The data was obtained from the clinical database of our hospital. Statistical analysis was performed using IBM SPSS Statistics 27. Propensity score matching was used for matching OE patients to HMIE patients. The calculation of propensity scores was performed by logistic regression with the matching criteria as independent covariables. The propensity scores were then used to manually match pairs between OE and HMIE patients. The maximum caliper between propensity scores was 0.096 and the median caliper was 0.0056. Matching criteria were gender, age, preoperative BMI, American Society of Anesthesiologists (ASA) classification, location of the tumor in the esophagus, and the type of neoadjuvant treatment received. For statistical testing we used Mann-Whitney U tests for continuous and Fisher’s exact tests for categorical variables. To illustrate associations between possible risk factors and certain outcomes like complications we performed logistic regression. To obtain double robustness with identification of confounders we conducted a multivariate forward stepwise regression in cases where prior tests showed statistically significant differences.

## 3. Results

### 3.1. Patient Characteristics

Between January 2010 and August 2022, a total of 174 patients underwent Ivor Lewis esophagectomy at the University department for General and Visceral surgery, Klinikum Oldenburg. Among these, 50 patients received HMIE, and the rest were treated with OE (*n* = 124). After matching, the median age of the study population was 64 years in the OE group (Range: 32–81 years) and 64.5 years in the HMIE group (Range: 41–83 years). Median BMI was 25.50 in the OE group (Range: 18.00–39.00) and 26.0 in the HMIE group (Range: 19.00–37.00). Patients were ASA classified preoperatively. The distribution of ASA classification was comparable between groups, with the majority being classified as ASA II and III (OE 94.00%, HMIE 98.00%). About half of the patients received no neoadjuvant treatment (OE 48.00%, HMIE 50.00%), whereas the predominant rest was treated with either chemotherapy (OE 40.00%, *n* = 20; HMIE 36.00%, *n* = 18) or radiochemotherapy (OE 12.00%, *n* = 6; HMIE 12.00%, *n* = 6) prior to surgery. One patient of the OE group received endoscopic submucosal dissection. Most cancers were located in the lower third of the esophagus (OE and HMIE 84%, *n* = 42). The most frequent comorbidities were cardiac, e.g., hypertension or history of myocardial infarction (OE 68.00%, *n* = 34; HMIE 54.00%, *n* = 27), followed by pulmonary comorbidities such as allergic asthma or COPD (OE 16.00%, *n* = 8; HMIE 16.00%, *n* = 8). A third of the OE patients (32.00%, *n* = 16) and almost half of the HMIE patients (46.00%, *n* = 23) had a history of smoking, while excessive alcohol consumption was only reported by a few patients (OE 6.00%, *n* = 3; HMIE 10%, *n* = 5). Further patient and tumor characteristics are listed in Table 1.

### 3.2. Surgical Results

The exact surgical results are displayed in Table 2. The oncological results in the form of the number of harvested lymphnodes (OE Median 23.50; HMIE Median 21.00) and cases with negative tumor margin (R0-resection: OE 98.00%, *n* = 49; HMIE 100%, *n* = 50) were comparable across the groups. Only a few patients needed RBC transfusion perioperatively (OE 12%, *n* = 6; HMIE 2.00%, *n* = 1) and there was no significant difference between the groups (*p* = 0.112), but the number of RBCs that needed to be applied per patient was significantly higher in the OE group than in the HMIE group (OE Median 0.00, Maximum 6.00; HMIE Median 0.00, Maximum 1.00; *p* = 0.041). Hospital stay was significantly shorter for HMIE patients (OE Median 23.00 days; HMIE 16.50 days; *p* = 0.004). Furthermore, OE patients stayed almost twice as long on the ICU compared to HMIE patients (OE 5.50 days; HMIE 3.00 days; *p* = 0.003).

### 3.3. Perioperative Morbidity and Mortality

Half of the patients had at least one complication (*n* = 50). However, complications were significantly more common in the OE group (OE 70.00%, *n* = 35; HMIE 30%, *n* = 15; *p* < 0.001). Categorized for severity of complications according to the Clavien Dindo classification, severe complications (Clavien Dindo III–V) occurred in 48% of the OE patients (*n* = 24), whereas only in 16% of HMIE patients (*n* = 8). This difference was also statistically highly significant (*p* < 0.001). A chi-square test was performed to test for the influence of complication variables on the overall complication rate. All associations were statistically significant and the φ coefficient for effect size demonstrated the strongest influence for pulmonary complications (χ^2^(1) = 44.444, *p* ≤ 0.001, φ = 0667), followed by pneumonia alone (χ^2^(1) = 41.360, *p* ≤ 0.001, φ = 0.643) (see Appendix D, Table A4). These were also the most common complications, and though not statistically significant, were more common after OE than after HMIE (pulmonary complications OE 46%, HMIE 26%, *p* = 0.060; pneumonia OE 38%, HMIE 26%, *p* = 0.284). Nine patients died perioperatively after a median of 19 days, ranging from 7 to 82 postoperative days (OE 14.0%, *n* = 7; HMIE 4.0%, *n* = 2; *p* = 0.160). Anastomotic leakage occurred in a total of 16 patients of the matched study population. A statistically significant difference between the groups could not be proven (OE 22.0%, *n* = 11; HMIE 10.0%, *n* = 5; *p* = 0.171). The anastomotic leakages were categorized in accordance with the classification of the Esophagectomy Complications Consensus Group’s (ECCG) classification. Anastomotic leakages that required surgical therapy (ECCG III) occurred in five patients after OE, while not at all in the HMIE group, but the difference failed to be statistically significant (*p* = 0.056). Further morbidity data is listed in Table 3.

### 3.4. Risk Factor Analysis

Univariate testing with Fisher’s exact test revealed a significant difference between HMIE and OE patients regarding the occurrence of severe complications (Clavien Dindo III–V) (*p* = 0.001). The OR for HMIE compared to OE was 0.206 (95% CI 0.081–0.527). In order to test for confounding factors, a multivariate forward stepwise regression was conducted, where surgical technique was confirmed to be an independent risk factor for severe complications. Furthermore, Fisher’s test showed significant differences between ASA categories I/II and III/IV in relation to perioperative death (*p* = 0.034; OR 1.212; 95% CI 0.521–2.822). In the multivariate regression model, cardiac comorbidities were also significant risk factors for death (*p* = 0.037). Regarding the occurrence of any complications, OE (univariate *p* = 0.001, multivariate *p* < 0.001; OR 0.206; 95% CI 0.081–0.527) and cardiac comorbidities (univariate *p* = 0.040; multivariate *p* = 0.012; OR 2.571; 95% CI 1.122–5.895) could be identified as independent risk factors. No risk factors could be identified for pulmonary complications in univariate analysis. However, multivariate stepwise forward regression proved the differences between OE and HMIE groups (46%/26%; multivariate *p* = 0.019) and patients with or without preexisting pulmonary morbidities (18%/54%; multivariate *p* = 0.033) to be statistically significant after adjusting for sex, age, BMI, ASA classification, histology, neoadjuvant treatment or not, smoking status, cardiac comorbidities, diabetes mellitus, and alcohol abuse.

The detailed risk factor analysis for outcomes that were shown to have statistically significant risk factors are displayed in Table 4 and Table 5.

As mentioned above, Mann-Whitney U tests also showed statistically significant differences between surgical techniques regarding duration of hospital (median 23 vs. 16.5 days; *p* = 0.004) and ICU stay (median 5.50 vs. 3 days; *p* = 0.003), as well as regarding the number of applied RBCs (maximum 6.0 vs. 1.0; *p* = 0.041). A multivariate forward stepwise analysis confirmed this while showing no significant confounding by sex, BMI ≥ 25, ASA III/ IV, neoadjuvant treatment, smoking, pulmonary or cardiac comorbidities, diabetes mellitus, or alcohol abuse (see Appendixe A, Appendixe B and Appendixe C, Table A1, Table A2 and Table A3).

## 4. Discussion

In a meta-analysis with a total of 2397 patients from 2019, Yang et al. showed comparable results of HMIE and OE regarding the number of harvested lymphnodes [12]. This was also true for our study population, which not only showed similar numbers of harvested lymphnodes, but also almost the complete number of patients being R0-resected in both groups (OE 98%, HMIE 100%). These findings also go in line with other retrospective and prospective studies [6,13,14].

HMIE is undoubtedly on the rise in comparison to the open method. The aim of this study was to compare HMIE and OE regarding perioperative and oncological results and with that, to analyze whether HMIE could hold up to the expectations derived from other gastrointestinal procedures where advantages of laparoscopic compared to conventional open procedures have been shown numerous times [15,16]. Some studies have already shown similarly significant differences between HMIE and OE and the results are promising.

In our study we were able to confirm a lower overall postoperative complication rate for HMIE compared to OE, and demonstrated an 82% higher risk for the occurrence of complications for the OE group, while the overall complication rate was comparable to other studies with a similar number of patients [6,8]. Furthermore, the risk to develop severe complications, which were categorized by a Clavien Dindo classification of III–V, was 80% higher in the OE group than in the HMIE group. Several studies showed a high pulmonary complication rate for all esophagectomy approaches, and it was frequently shown that this rate was significantly lower for HMIE patients in comparison to OE [17,18]. We were not able to support this observation alone with statistically significant tests, but our data showed a clear tendency in the same direction, as pulmonary complications were observed in 46% of OE patients and only in 26% of HMIE patients (*p* = 0.06). Additionally, we could confirm the above mentioned assumption after adjustment for sex, age, BMI, ASA classification, histology, neoadjuvant treatment or not, smoking status, cardiac comorbidities, diabetes mellitus, and alcohol abuse (*p* = 0.019). We were also able to demonstrate a significant influence of pulmonary comorbidities on the development of pulmonary complications after any kind of esophagectomy in said adjusted setting (*p* = 0.033). The fact that pulmonary complications were significantly more common after OE than after HMIE raises the question of its cause, since all patients received the same kind of thoracotomy. However, the difference between HMIE and OE was the abdominal part, and considering that several studies have shown a higher rate of postoperative pneumonia after laparotomy compared to laparoscopy, the higher number of pulmonary complications in this study’s OE group could be partially caused by the abdominal approach [19,20]. Another possible explanation could be the difference in operating time, and with that the longer duration of mechanical ventilation, which has also been shown to be an independent risk factor for the development of postoperative pneumonia [21].

The rate of anastomotic leakages was higher after OE than after HMIE (OE 22%, HMIE 10%, *p* = 0.171). Although not statistically significant, the difference between rates of anastomotic leakages between the groups is remarkable. Known risk factors for said leakages, such as BMI > 30 kg/m^2^ or < 18.5 kg/m^2^, diabetes, or a smoking history are distributed equally between OE and HMIE groups, so the different frequencies cannot be explained by these [22]. However, a possible contribution could be a longer impaired circulation during the surgery since the groups are matched as homogeneously as possible, but we detected a difference in the duration of surgery and the needed number of RBCs [23,24]. In general, the results regarding anastomotic leakage rates are heterogenous throughout studies. While some report lower rates for OE compared to HMIE, other studies also show appreciably higher rates of anastomotic leakages after OE compared to HMIE [6,13]. Finally, a 2019 meta-analysis by Yang et al., including 17 studies and 2397 patients, also showed no difference between HMIE and OE (OR 0.95, 95% CI 0.67–1.35) [12]. Our results go in line with the literature here, since in our study the difference between anastomotic leakage rates is not statistically significant (*p* = 0.171).

Besides overall and pulmonary complications, we differentiated between severe complications and minor complications according to the Clavien Dindo classification. We considered complications classified Clavien Dindo III and above as severe, meaning at least the requirement of surgical, endoscopic, or radiological intervention [25]. In our study population these requirements were met by 48% of the OE group and 16% of HMIE patients with an Odds ratio of 0.206 (95% CI 0.081–0.527), which meant a large increase in risk of almost 80% for OE patients.

We were also able to show a significantly shorter hospital and ICU stay, as well as a significantly smaller number of applied RBCs for HMIE compared to OE patients. Since these are rather cost-relevant factors it would be interesting to perform a dedicated cost analysis of both procedures to see whether one is superior in this regard.

## 5. Limitations

This study had some limitations. The most important being the retrospective approach with all its weaknesses, including possible information bias, as it is not possible to fully ensure complete data in medical records. Another weakness is the single center setting that limited the size of our study population. Due to the matching procedure, additional patients had to be excluded from the analysis, which on one hand led to a robust handling of confounders, but on the other hand additionally reduced the number of patients.

Improvement of ICU management over time could have led to a lower overall complication rate in the HMIE group, of which the majority of patients received their surgery more recently than OE patients.

An analysis of risk factors was only conducted for the overall patient collective. To achieve statistically significant effects for analyses of subgroups a larger number of patients would be necessary. It would be interesting to know if the different surgical techniques come with different risk factors for certain complications.

One further limitation certainly is the restricted follow-up time. Our data collection was restricted to the hospital stay in which the operation took place. We were able to interpret the immediate oncological results and mortality, but long-term outcome, especially regarding recurrence rate or survival rates, could not be analyzed.

## 6. Conclusions

Our study clearly demonstrated reduced postoperative morbidity and in hospital-mortality for HMIE in comparison with OE. Not only were overall complications less likely to occur, but they were less severe than in OE cases. Furthermore, patients stayed significantly shorter in the ICU and were discharged faster when operated on with the HMIE approach. All this confirms the beneficial effects found in other studies and for other minimally-invasive surgical techniques of esophagectomy or other surgeries of the gastrointestinal tract.

Summed up, HMIE is a feasible technique that significantly decreases morbidity while ensuring equivalently good oncological resection compared to OE. HMIE should be performed whenever applicable for patients and surgeons.

## Figures and Tables

**Table 1:** Clinicopathological features in association with surgical technique.

		Open (*n* = 50)			Hybrid (*n* = 50)			
		*n*	%	Median	*n*	%	Median	*p*
Sex	Male	41	82.0%		40	80.0%		1.000
	Female	9	18.0%		10	20.0%		1.000
Age in years				64.00			64.50	0.373
Preoperative BMI				25.50			26.00	0.371
ASA-Classification	ASA I	0	0.0%		0	0.0%		
	ASA II	26	52.0%		21	42.0%		0.423
	ASA III	21	42.0%		28	56.0%		0.230
	ASA IV	3	6.0%		1	2.0%		0.617
	ASA V	0	0.0%		0	0.0%		
Neoadjuvant Treatment	None	24	48.0%		25	50.0%		1.000
	Chemotherapy	20	40.0%		18	36.0%		0.837
	Radio-Chemotherapy	6	12.0%		6	12.0%		1.000
	Endoscopic Submucosal Dissection	0	0.0%		1	2.0%		1.000
Postoperative UICC-Classification	0	7			8			1.000
	IA	14			12			0.820
	IB	2			9			0.051
	IIA	4			7			0.525
	IIB	9			0			0.003
	III	14			12			0.820
	IV	0			2			0.495
Histology of Tumor	No malignancy	1	2.0%		0	0.0%		1.000
	Adenocarcinoma	40	81.6%		43	86.0%		0.595
	Squamous Cell Carcinoma	8	16.3%		7	14.0%		1.000
Location of Tumor	Lower third	42	84.0%		42	84.0%		1.000
	Middle third	8	16.0%		7	14.0%		1.000
	Upper third	0	0.0%		1	2.0%		1.000
Smoking		16	32.0%		23	46.0%		0.218
Alcohol abuse		3	6.0%		5	10.0%		0.715
Diabetes mellitus		5	10.0%		4	8.0%		1.000
Cardiac Comorbidities		34	68.0%		27	54.0%		0.218
Pulmonary Comorbidities		8	16.0%		8	16.0%		1.000

Statistical testing was performed with Fisher’s exact test resp. Mann-Whitney U test. BMI = Body Mass Index, ASA = American Society of Anesthesiologists-Classification, UICC = Union Internationale Contre le Cancer-classification.

**Table 2:** Surgical outcome in association with surgical technique.

		Open (*n* = 50)				Hybrid (*n* = 50)				
		Median	Maximum	Minimum	*n* (%)	Median	Maximum	Minimum	*n* (%)	*p*
Number of Harvested Lymphnodes		23.50	41.00	12.00		21.00	38.00	13.00		0.666
Duration of Surgery		288.00	482.00	127.00		225.50	337.00	122.00		0.243
Tumor Margin	R0				49 (98.00)				50 (100.00)	1.000
	R1				0				0	
	R2				1 (2.00)				0	1.000
Required RBC Transfusion					6 (12.00)				1 (2.00)	0.112
Number of RBCs		0.00	6.00	0.00		0.00	1.00	0.00		0.041
Hospital Stay (days)		23.00	123.00	7.00		16.50	142.00	9.00		0.004
IMC Stay (days)		4.00	15.00	0.00		2.00	8.00	0.00		0.061
ICU Stay (days)		5.50	71.00	2.00		3.00	117.00	1.00		0.003

Statistical testing was performed with Fisher’s exact test resp. Mann-Whitney U test. RBC = red blood cells units, IMC = intermediate care unit, ICU = intensive care unit.

**Table 3:** Postoperative Morbidity and Mortality.

	Open (*n* = 50)		Hybrid (*n* = 50)		
	*n*	%	*n*	%	*p*
Any Complication	35	70.0%	15	30.0%	<0.001
Clavien Dindo I-II	26	52.0%	42	84.0%	0.001
Clavien Dindo III-V	24	48.0%	8	16.0%	0.001
Anastomotic Leakage	11	22.0%	5	10.0%	0.171
ECCG I	0	0%	0	0%	
ECCG II	6	12%	5	10%	1.000
ECCG III	5	10%	0	0%	0.056
Pulmonary Embolism	3	6.0%	1	2.0%	0.617
Pulmonary Complication	23	46.0%	13	26.0%	0.060
Pneumonia	19	38.0%	13	26.0%	0.284
ARDS	6	12.0%	2	4.0%	0.269
Perioperative Death	7	14.0%	2	4.0%	0.160

Statistical testing was performed with Fisher’s exact test. ECCG = Esophagectomy Complications Consensus Group classification, ARDS = Acute Respiratory Distress Syndrome.

**Table 4:** Risk factor analysis of overall complications and severe complications (Clavien Dindo III–V).

	Any Complication				Clavien Dindo III–V			
	OE/ HMIE (%)	*p* ^a^	OR ^a^ (95% CI)	*p* ^b^	OE/HMIE (%)	*p* ^a^	OR ^a^ (95% CI)	*p* ^b^
Surgical Technique (OE/HMIE)	70/30	<0.001	0.184 (0.078–0.432)	< 0.001	48/16	0.001	0.206 (0.081–0.527)	<0.001
Sex (male/female)	86/14	0.308		0.103	52/12	1.000		0.483
Age (<65 years/≥65 years)	54/46	0.689		0.277	36/28	0.524		0.238
BMI (≥25/<25)	60/40	1.000		0.420	38/26	1.000		0.479
ASA (I–II/ III–IV)	40/60	0.229		0.082	28/36	0.674		0.329
Histology (adeno/SCC)	40/16	0.595		0.215	54/10	1.000		0.411
Neoadjuvant treatment (any/none)	48/52	1.000		0.422	36/28	0.524		0.306
Smoking (yes/no)	34/66	0.412		0.155	26/38	0.829		0.260
Pulmonary Comorbidities (yes/no)	20/80	0.414		0.140	12/52	0.771		0.057
Cardiac Comorbidities (yes/no)	72/28	0.040	2.571 (1.122–5.895)	0.012	42/22	0.661		0.331
Diabetes mellitus (yes/no)	14/86	0.160		0.041	10/54	0.140		0.402
Alcohol abuse (yes/no)	4/96	0.269		0.072	4/60	1.000		0.238

^a^ Univariate analysis including Fisher’s exact test. ^b^ Multivariate stepwise forward regression.

**Table 5:** Risk factor analysis of pulmonary complications and perioperative death.

	Pulmonary Complication				In-Hospital Death			
	OE/HMIE (%)	*p* ^a^	OR ^a^ (95% CI)	*p* ^b^	OE/HMIE (%)	*p* ^a^	OR ^a^ (95% CI)	*p* ^b^
Surgical Technique (OE/HMIE)	46/26	0.060		0.019	14/4	0.160		0.041
Sex (male/ female)	60/12	0.793		0.330	16/2	1.000		0.266
Age (<65 years/≥65 years)	40/32	0.537		0.250	4/14	0.089		0.036
BMI (≥25/<25)	44/28	0.833		0.375	12/6	0.733		0.314
ASA (I–II/III–IV)	30/42	0.532		0.214	2/16	0.034	1.212 (0.521–2.822)	0.012
Histology (adeno/SCC)	60/12	1.000		0.474	18/0	0.351		0.079
Neoadjuvant treatment (any/none)	36/36	1.000		0.441	8/10	0.738		0.342
Smoking (yes/no)	22/50	0.209		0.099	8/10	0.733		0.364
Pulmonary Comorbidities (yes/no)	18/54	0.089		0.033	4/14	0.633		0.299
Cardiac Comorbidities (yes/no)	50/22	0.209		0.099	16/2	0.086		0.037
Diabetes mellitus (yes/no)	6/66	1.000		0.432	4/14	0.186		0.075
Alcohol abuse (yes/no)	4/68	0.707		0.252	0/18	1.00		0.179

^a^ Univariate analysis including Fisher’s exact test. ^b^ Multivariate stepwise forward regression.

## Data Availability

Supporting results can be found in the Appendixe A, Appendixe B, Appendixe C and Appendixe D, Table A1, Table A2, Table A3 and Table A4 of this paper. The data presented in this study are available on request from the corresponding author. The data are not publicly available due to data protection restrictions of our institution.

## Appendix A

**Table A1:** Step 1 of multivariate stepwise forward linear regression.

		Regression Coefficient B	Std Error	Wald	df	*p*	Exp (B)
Step 1 ^a^	ICU Stay (days)	0.039	0.028	1.947	1	0.163	1.040
	Hospital Stay (days)	−0.045	0.021	4.601	1	0.032	0.956
	Number of RBCs	−1.455	0.819	3.156	1	0.076	0.233
	Constant	1.030	0.426	5.856	1	0.016	2.801

^a^ In Step 1 inserted variables: ICU Stay (days), Hospital Stay (days), Number of RBCs.

## Appendix B

**Table A2:** Step 2 of multivariate stepwise forward linear regression.

		Regression Coefficient B	Std Error	Wald	df	*p*	Exp (B)
Step 2 ^a^	ICU Stay (days)	0.036	0.028	1.597	1	0.206	1.037
	Hospital Stay (days)	−0.043	0.021	4.395	1	0.036	0.958
	Number of RBCs	−1.617	0.840	3.700	1	0.054	0.199
	Gender Female	−0.050	0.548	0.008	1	0.927	0.951
	BMI >/= 25	0.000	0.445	0.000	1	0.999	1.000
	ASA 3/4	0.607	0.443	1.876	1	0.171	1.835
	Constant	0.719	0.565	1.620	1	0.203	2.052

^a^ In Step 2 inserted variables: Gender Female, BMI >/= 25, ASA 3/4.

## Appendix C

**Table A3:** Step 3 of multivariate stepwise forward linear regression.

		Regression Coefficient B	Std Error	Wald	df	*p*	Exp (B)
Step 3 ^a^	ICU Stay (days)	0.030	0.031	0.945	1	0.331	1.031
	Hospital Stay (days)	−0.043	0.022	3.821	1	0.051	0.958
	Number of RBCs	−1.656	0.953	3.019	1	0.082	0.191
	Gender Female	−0.068	0.567	0.014	1	0.904	0.934
	BMI >/= 25	0.178	0.465	0.147	1	0.701	1.195
	ASA 3/4	0.813	0.499	2.658	1	0.103	2.256
	Neoadjuvant Treatment	0.203	0.329	0.380	1	0.537	1.225
	Smoking	0.418	0.475	0.774	1	0.379	1.518
	Pulmonary Comorbidities	0.437	0.688	0.403	1	0.526	1.547
	Cardiac Comorbidities	−0.859	0.503	2.925	1	0.087	0.423
	Diabetes mellitus	−0.144	0.837	0.030	1	0.863	0.866
	Alcohol abuse	0.284	0.840	0.114	1	0.735	1.328
	Constant	0.687	0.700	0.964	1	0.326	1.989

^a^ In Step 3 inserted variables: Gender Female, BMI >/= 25, ASA 3/4, Neoadjuvant Treatment, Smoking, Pulmonary Comorbidities, Cardiac Comorbidities, Diabetes mellitus, Alcohol abuse.

## Appendix D

**Table A4:** Chi-square test. Association between overall complications and individual complications.

	Pearson-Chi-Square Value	df	*p*	φ
Anastomotic leakage	19.048	1	<0.001	0.436
Pneumonia	41.360	1	<0.001	0.643
ARDS	8.696	1	0.003	0.295
Pulmonary complication	44.444	1	<0.001	0.667
Pulmonary embolism	4.167	1	0.041	0.204
Death	5.983	1	0.014	0.245

ARDS = Acute respiratory distress syndrome.

## References

[1] AWMF and Leitlinienprogramm Onkologie (2021). S3-Leitlinie Diagnostik und Therapie der Plattenepithelkarzinome und Adenokarzinome des Ösophagus: Version 3.1., AWMF-Registernummer: 021/023OL.

[2] Moehler M., Al-Batran S.E., Andus T., Anthuber M., Arends J., Arnold D., Aust D., Baier P., Baretton G., Bernhardt J. (2011). German S3-guideline “Diagnosis and treatment of esophagogastric cancer. Z. Fur Gastroenterol..

[3] Schröder W., Gisbertz S.S., Voeten D.M., Gutschow C.A., Fuchs H.F., van Berge Henegouwen M.I. (2021). Surgical Therapy of Esophageal Adenocarcinoma-Current Standards and Future Perspectives. Cancers.

[4] van Workum F., Klarenbeek B.R., Baranov N., Rovers M.M., Rosman C. (2020). Totally minimally invasive esophagectomy versus hybrid minimally invasive esophagectomy: Systematic review and meta-analysis. Dis. Esophagus.

[5] Harriott C.B., Angeramo C.A., Casas M.A., Schlottmann F. (2022). Open versus hybrid versus totally minimally invasive Ivor Lewis esophagectomy: Systematic review and meta-analysis. J. Thorac. Cardiovasc. Surg..

[6] Mariette C., Markar S.R., Dabakuyo-Yonli T.S., Meunier B., Pezet D., Collet D., D’Journo X.B., Brigand C., Perniceni T., Carrère N. (2019). Hybrid Minimally Invasive Esophagectomy for Esophageal Cancer. N. Engl. J. Med..

[7] Nuytens F., Dabakuyo-Yonli T.S., Meunier B., Gagnière J., Collet D., D’Journo X.B., Brigand C., Perniceni T., Carrère N., Mabrut J.Y. (2021). Five-Year Survival Outcomes of Hybrid Minimally Invasive Esophagectomy in Esophageal Cancer: Results of the MIRO Randomized Clinical Trial. JAMA Surg..

[8] Biere S.S., van Berge Henegouwen M.I., Maas K.W., Bonavina L., Rosman C., Garcia J.R., Gisbertz S.S., Klinkenbijl J.H., Hollmann M.W., De Lange E.S. (2012). Minimally invasive versus open oesophagectomy for patients with oesophageal cancer: A multicentre, open-label, randomised controlled trial. Lancet.

[9] Van der Wilk B.J., Hagens E.R., Eyck B.M., Gisbertz S.S., van Hillegersberg R., Nafteux P., Schröder W., Nilsson M., Wijnhoven B.P., Lagarde S.M. (2022). Outcomes after totally minimally invasive versus hybrid and open Ivor Lewis oesophagectomy: Results from the International Esodata Study Group. Br. J. Surg..

[10] AWMF and Leitlinienprogramm Onkologie (2018). S3-Leitlinie Diagnostik und Therapie der Plattenepithelkarzinome und Adenokarzinome des Ösophagus.: Version 2.0. https://www.leitlinienprogramm-onkologie.de/leitlinien/oesophaguskarzinom/.

[11] Porschen R., Buck A., Fischbach W., Gockel I., Görling U., Grenacher L., Hollerbach S., Hölscher A., Körber J., Messmann H. (2015). S3-Leitlinie Diagnostik und Therapie der Plattenepithelkarzinome und Adenokarzinome des Ösophagus (Langversion 1.0—September 2015, AWMF-Registernummer: 021/023OL). Z. Fur Gastroenterol..

[12] Yang J., Chen L., Ge K., Yang J.L. (2019). Efficacy of hybrid minimally invasive esophagectomy vs. open esophagectomy for esophageal cancer: A meta-analysis. World J. Gastrointest. Oncol..

[13] Glatz T., Marjanovic G., Kulemann B., Sick O., Hopt U.T., Hoeppner J. (2017). Hybrid minimally invasive esophagectomy vs. open esophagectomy: A matched case analysis in 120 patients. Langenbeck’s Arch. Surg..

[14] Bailey L., Khan O., Willows E., Somers S., Mercer S., Toh S. (2013). Open and laparoscopically assisted oesophagectomy: A prospective comparative study. Eur. J. Cardio-Thorac. Surg..

[15] Lacy A.M., García-Valdecasas J.C., Delgado S., Castells A., Taurá P., Piqué J.M., Visa J. (2002). Laparoscopy-assisted colectomy versus open colectomy for treatment of non-metastatic colon cancer: A randomised trial. Lancet.

[16] Van der Pas M.H., Haglind E., Cuesta M.A., Fürst A., Lacy A.M., Hop W.C., Bonjer H.J. (2013). Laparoscopic versus open surgery for rectal cancer (COLOR II): Short-term outcomes of a randomised, phase 3 trial. Lancet. Oncol..

[17] Bjelovic M., Babic T., Spica B., Gunjic D., Veselinovic M., Trajkovic G. (2016). Could hybrid minimally invasive esophagectomy improve the treatment results of esophageal cancer?. Eur. J. Surg. Oncol..

[18] Briez N., Piessen G., Claret A., Triboulet J., Mariette C. (2010). Is minimally invasive œsophagectomy for cancer decreasing pulmonary complications?. Results from a case-control study. J. Clin. Oncol..

[19] Sparn M.B., Widmann B., Pietsch U., Weitzendorfer M., Warschkow R., Steffen T. (2021). Risk factors and outcomes of postoperative aspiration pneumonia in abdominal surgery patients: An exact matching and weighting analysis. Surgery.

[20] Baba H., Tokai R., Hirano K., Watanabe T., Shibuya K., Hashimoto I., Hojo S., Yoshioka I., Okumura T., Nagata T. (2020). Risk factors for postoperative pneumonia after general and digestive surgery: A retrospective single-center study. Surg. Today.

[21] Charles M.P., Kali A., Easow J.M., Joseph N.M., Ravishankar M., Srinivasan S., Kumar S., Umadevi S. (2014). Ventilator-associated pneumonia. Australas. Med. J..

[22] Fabbi M., Hagens E.R.C., van Berge Henegouwen M.I., Gisbertz S.S. (2021). Anastomotic leakage after esophagectomy for esophageal cancer: Definitions, diagnostics, and treatment. Dis. Esophagus.

[23] Welte M., Saugel B., Reuter D.A. (2020). Perioperative blood pressure management: What is the optimal pressure?. Anaesthesist.

[24] Chughtai M., Gwam C.U., Mohamed N., Khlopas A., Newman J.M., Khan R., Nadhim A., Shaffiy S., Mont M.A. (2017). The Epidemiology and Risk Factors for Postoperative Pneumonia. J. Clin. Med. Res..

[25] Dindo D., Cuesta M., Bonjer H.J. (2014). The Clavien–Dindo Classification of Surgical Complications. Treatment of Postoperative Complications after Digestive Surgery.

